# Effect of PPARγ Inhibition on Bone in Aged Animals

**DOI:** 10.1093/geroni/igaa057.408

**Published:** 2020-12-16

**Authors:** Xingming Shi, Kehong Ding, Yun Su, Carlos Isales

**Affiliations:** 1 Augusta University, Augusta, Georgia, United States; 2 augusta university, Augusta, Georgia, United States

## Abstract

Aging is accompanied by bone loss and marrowfat accumulation. PPARg is a key factor regulating adipocyte differentiation. Marrowfat secretes large quantities of factors including adipokines, cytokines and chemokines that contribute to bone loss. We hypothesized that inhibition of PPARg would reduce adipocytes and adipose-generated inflammatory factors, and thus decrease aging-induced bone loss. To test this hypothesis, we treated young (6mo) and old (23mo) female C57BL/6 mice with a PPARg antagonist GW9662 (1mg/kg body weight, IP injection for 6wks) and examined the effects on bone, immune cell lineage skewing, and the expression of inflammation related genes. DXA analysis data showed that GW treatment had no effect on vertebral and femoral BMD and BMC in young or old mice. u-CT analysis showed that GW treatment reduced BV/TV in old mice but had no effect in young mice. FACS analysis data showed that GW treatment significantly increased lymphoid (CD3) and decreased myeloid (CD11b) lineage cells only in young mice. GW treatment significantly decreased CD4 and CD8 T cell subpopulations in young mice, but had no effect in old mice. Interestingly, Nanostring analysis of inflammation genes revealed an opposite effects of GW treatment in young vs. old mice, i.e., genes whose expression were downregulated by GW in bone cells of the young mice were upregulated in the old mice. These results suggested that systemic inhibition of PPARg, while enhancing CD3 and reducing CD11b cell populations in the bone marrow of young mice, may have a negative effect on bone architecture in old mice.

